# Evaluation du risque de mort subite lié à lacardiomyopathie hypertrophique à Dakar

**DOI:** 10.5830/CVJA-2017-010

**Published:** 2018

**Authors:** Antoine Sarr Simon, Dodo Boubacar, Babaka Kana, Aw Fatou, Bodian Malick, Bamba Ndiaye Mouhamadou, Kane Adama, Diao Maboury, Abdou Ba Serigne

**Affiliations:** Department of Cardiology, Teaching Hospital Aristide Le Dantec, Dakar, Senegal; Department of Cardiology, Teaching Hospital Aristide Le Dantec, Dakar, Senegal; Department of Cardiology, Teaching Hospital Aristide Le Dantec, Dakar, Senegal; Department of Cardiology, Teaching Hospital Aristide Le Dantec, Dakar, Senegal; Department of Cardiology, Teaching Hospital Aristide Le Dantec, Dakar, Senegal; Department of Cardiology, Teaching Hospital Aristide Le Dantec, Dakar, Senegal; Department of Cardiology, Teaching Hospital Aristide Le Dantec, Dakar, Senegal; Department of Cardiology, Teaching Hospital Aristide Le Dantec, Dakar, Senegal; Department of Cardiology, Teaching Hospital Aristide Le Dantec, Dakar, Senegal

**Keywords:** cardiomyopathie hypertrophique (CMH), score, mort subite, DAI, hypertrophic cardiomyopathy, HCM, sudden death, implantable cardioverter defibrillator, score

## Abstract

**Objectifs:**

La cardiomyopathie hypertrophique (CMH) est l’une des principales causes de mort subite (MS) du sujet jeune, notamment chez le sportif de moins de 35 ans. Le niveau de risque est variable et nécessite d’être évalué afin d’adopter une stratégie préventive adaptée. Nous avons entrepris ce travail dans le but d’évaluer le risque de survenue de mort subite dans une population de CMH à Dakar.

**Méthode:**

Il s’agissait d’une étude transversale et descriptive menée à la clinique cardiologique de l’hôpital Aristide Le Dantec de Dakar du 1er Janvier 2014 au 30 Juin 2015. Nous avions évalué sur le plan clinique et paraclinique les facteurs de risque de mort subite et utilisé le score en ligne de l’European Society of Cardiology (ESC) pour le calcul de ce risque. La population étudiée était constituée de patients porteurs de CMH diagnostiquée, suivis dans ledit service.

**Résultats:**

Nous avions retrouvé un âge moyen des patients de 53.25 ans et il y avait une prédominance masculine (sexratio de 1.66). La syncope inexpliquée était retrouvée chez 2 patients et 2 autres avaient des antécédents de survenue de mort subite dans leurs familles à des âges de 50 ans et 55 ans. L’hypertrophie septale maximale était en moyenne de 20.9 mm. Quatorze patients présentaient une dilatation auriculaire gauche. Sept patients présentaient une obstruction intra-ventriculaire gauche. Selon le score ESC, 1 patient avait un haut risque de survenue de mort subite dans les 5 ans, 3 un risque intermédiaire et 13 un risque faible. Le sport de compétition était proscrit, 13 patients étaient sous traitement médical, 1 avait eu un défibrillateur automatique implantable (DAI) et 2 n’étaient sous aucun traitement.

**Conclusion:**

Notre travail a mis en exergue une prédominance de risque faible et intermédiaire de mort subite à 5 ans. Le haut risque existait dans un cas.

## Abstract (en)

**Aim:**

The aim of this study was to assess of the risk of sudden death in a population of hypertrophic cardiomyopathy patients in Dakar.

**Methods:**

This was a transverse study at the cardiology clinic of Aristide Le Dantec Hospital from January 2014 to June 2015. We used the European Society of Cardiology risk score to calculate this risk.

**Results:**

The average age of patients was 53 years. Unexplained syncope was found in two patients and two others had a family history of sudden death. The septal hypertrophy average was 20.9 mm. Seven patients had left intraventricular obstruction. One patient had a high risk of sudden death, three had intermediate risk and 13 had low risk. Competitive sport was not allowed, 13 patients were under medical treatment, one had an implantable cardioverter defibrillator and two had no treatment.

**Conclusion:**

Our study highlighted a low and intermediate risk of the occurrence of sudden death at five years. One patient had a high risk of sudden death.

La cardiomyopathie hypertrophique (CMH) est la plus fréquente des maladies cardiaques d’origine génétique dont la transmission se fait par le mode autosomique dominant.^1^ Il s’agit d’une affection primitive se caractérisant par une hypertrophie pariétale du ventricule gauche, en règle asymétrique, le plus souvent septale, et s’accompagnant inconstamment d’obstruction ventriculaire.^2^,^3^

Elle représente l’une des principales causes de mort subite (MS) du sujet jeune, notamment chez le sportif de moins de 35 ans et, est rencontrée dans environ 0.5% des patients référés à un laboratoire d’échocardiographie en l’absence de toute sélection préalable.^2^ Il existe une grande hétérogénéité dans l’expression et l’évolution de la maladie, et la majorité des patients reste asymptomatique ou paucisymptomatique pendant très longtemps.^4^

La mort subite (MS) demeure la complication redoutée de la maladie par sa gravité et son caractère imprévisible. Elle peut constituer la première manifestation de la maladie, son incidence est d’environ 1% par an.^4^-^6^ Elle est habituellement en relation avec une tachyarythmie ventriculaire, le stimulus initial pouvant être variable (trouble du rythme supraventriculaire, chute excessive des résistances vasculaires à l’effort, ischémie d’effort, augmentation brutale du gradient intraventriculaire, troubles de conduction). Ce dernier survient volontiers au cours ou au décours immédiat d’un effort physique important.^5^,^6^

Le but de notre étude était d’évaluer le risque de survenue de mort subite chez les patients porteurs de CMH à Dakar, en procédant à des examens cliniques et paracliniques afin de proposer une prise en charge adaptée.

## Méthode

Il s’agissait d’une étude transversale et descriptive réalisée du 1er Janvier 2014 au 30 Juin 2015 à la clinique cardiologique de l’hôpital Aristide Le Dantec de Dakar. Le travail a inclus tous les patients porteurs de CMH vus en consultation (CMH connue) ou en laboratoire d’échocardiographie. Les critères diagnostiques à l’échocardiographie étaient une épaisseur de paroi ≥ 13 mm dans un contexte familial ou ≥ 15 mm en l’absence de contexte familial associée à un rapport septum interventriculaire/paroi postérieure > 1.3 (SIV/PP). Les patients qui présentaient une cause d’hypertrophie ventriculaire gauche (HVG) étaient exclus. Un dépistage familial était proposé et les nouveaux cas étaient inclus.

Les patients ont préalablement après consentement éclairé, accepté de répondre au questionnaire et de réaliser les examens paracliniques appropriés. Après examen clinique, tous les patients avaient eu un tracé électrocardiographique, une échocardiographie Doppler, un Holter ECG des 24 heures. L’échocardiographie utilisait une sonde d’imagerie de 3 à 7.0 MHz connecté à un système Vivid 7 Dimension’06 de General ElectricÒ. Afin de minimiser la variabilité entre les examens, tous les enregistrements échocardiographiques étaient effectués par le même médecin. Un test d’effort sur tapis roulant était fait chez tous les patients qui étaient aptes à marcher sur tapis.

Les facteurs de risque de survenue de MS ont été évalués et nous avions utilisé le score en ligne de l’ESC pour le calcul.^7^ Ce score est basé sur un algorithme (validé sur une cohorte de près de 3 000 patients) pour le calcul du risque de survenue d’une MS dans les 5 ans. Il prend en compte l’âge (ans), l’HVG maximale (mm), le pic d’obstruction sous aortique (mmHg) spontané ou au Valsalva, le diamètre antéro-postérieur atrial gauche (mm), l’antécédent de syncope inexpliquée (0/1), l’antécédent familial de mort subite < 40 ans ou à tout âge si rapportée à une CMH (0/1), la présence de tachycardie ventriculaire non soutenue au Holter ECG de 48 heures (0/1). Il permet de prendre une décision par rapport à la prévention primaire de la MS. Concernant les résultats, il y a trois possibilités. Ainsi, lorsque le score < 4% le risque est considéré comme faible ; entre 4–6% il était intermédiaire (et l’implantation d’un DAI peut être discutée); > 6% il était élevé et un DAI est formellement indiqué.

Le calcul du risque est possible en ligne sur le web sur le site de l’ESC,^7^ la formule est la suivante:

Probability_SCD at 5 years_ = 1 – 0.998^exp(Prognostic index)^ where prognostic index = [0.15939858 × maximal wall thickness (mm)] – [0.00294271 × maximal wall thickness^2^ (mm^2^)] + [0.0259082 × left atrial diameter (mm)] + [0.00446131 × maximal (rest/Valsalva) left ventricular outflow tract gradient (mm Hg)] + [0.4583082 × family history SCD] + [0.82639195 × NSVT] + [0.71650361 × unexplained syncope] – [0.01799934 × age at clinical evaluation (years)].

N.B. In HCM risk-SCD there was a non-linear relationship between the risk of SCD and maximum left ventricular wall thickness. This is accounted for in the risk prediction model by the inclusion of a quadratic term for maximum left ventricular wall thickness.

## Résultats

Au total, 16 patients étaient inclus. L’âge moyen était de 53.25 ans (27–79 ans). Huit patients avaient un âge compris entre 45 et 64 ans. Il y avait une prédominance masculine (sex ratio de 1.66).

En ce qui concerne les facteurs majeurs de MS à l’interrogatoire, une syncope était rapportée chez deux patients. Par ailleurs, deux autres avaient des antécédents familiaux de mort subite à 50 ans et 55 ans. Le tableau I résume les anomalies cliniques.

**Tableau I:** Anomalies cliniques retrouvées chez les patients porteurs d’une cardiomyopathie hypertrophique

*Anomalies cliniques*	*Nombre de personnes (n = 16)*
Mort subite familiale	2
Syncope	2
Palpitations	13
Dyspnée d’effort	13
Souffle systolique d’insuffisance mitrale	7
Souffle systolique éjectionnel	4

**Fig. 1:**
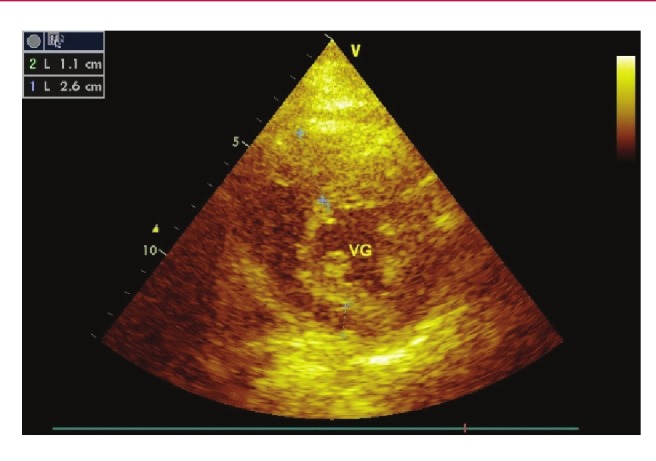
Image d’échographie cardiaque coupe parasternale petit axe d’un patient de 27 ans porteur de cardiomyopathie hypertrophique. Notez l’hypertrophie septale (26 mm) par rapport à la paroi postérieure (11 mm).

**Fig. 2:**
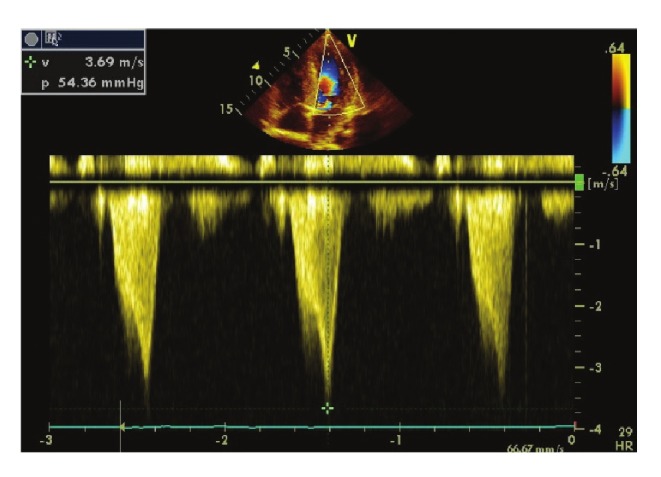
Image d’échographie–Doppler cardiaque coupe apicale 4 cavités d’un patient de 27 ans porteur d’une cardiomyopathie hypertrophique obstructive. On note la présence d’un gradient intra–ventriculaire gauche mesuré à 54.3 mmHg en forme de lame de sabre.

Sur le plan électrocardiographique, un cas de fibrillation atriale était noté. Une HVG était trouvée dans 11 cas. A l’échocardiographie Doppler, l’hypertrophie septale maximale était en moyenne de 20.9 mm (13.8–27 mm). Le rapport SIV/ PP était en moyenne de 2.2 (1.5–4.1) (Fig. 1). La surface de l’oreillette gauche était en moyenne de 21.3 cm^2^ (16–30 cm^2^). Le diamètre de l’oreillette gauche était en moyenne de 42.7 mm (36–51 mm). Quatorze patients présentaient une dilatation auriculaire gauche. Sept patients présentaient une obstruction intra-ventriculaire gauche (intra-VG). Cette obstruction était médio-ventriculaire dans quatre cas et intéressait la chambre de chasse dans trois cas. Le gradient maximal intra-ventriculaire gauche (intra-VG) était en moyenne de 31.8 mm Hg (13–54 mm Hg) (Fig. 2).

Le Holter ECG des 24 heures révélait des troubles du rythme ventriculaire chez trois patients. Un d’entre eux présentait des épisodes de tachycardie ventriculaire non soutenue après phénomène R/T. Ils étaient classés en hyperexcitabilité ventriculaire degrés I, IVB et V de Lown. Il y avait dans un cas, des troubles de la conduction à type d’alternance de bloc auriculo-ventriculaire (BAV) de 2ème degré Mobitz I et Mobitz II.

Dix patients avaient pu effectuer le test d’effort qui était maquillé pour huit d’entre eux déjà sous traitement. Les six autres n’ont pu le faire pour des raisons diverses comme l’âge avancé ou des séquelles d’AVC. Un patient avait présenté une mauvaise adaptation des chiffres tensionnels à l’effort avec la différence entre sa pression artérielle systolique (PAS) au maximum de l’effort (palier cinq) et celle au repos < à 20 mmHg. Une patiente avait présenté des nombreuses extrasystoles auriculaires avec apparition d’un PR court. Trois patients avaient présenté un sous-décalage non significatif du segment ST en latéral bas. Nous n’avions pas noté l’apparition de syncope lors du test d’effort.

**Fig. 3:**
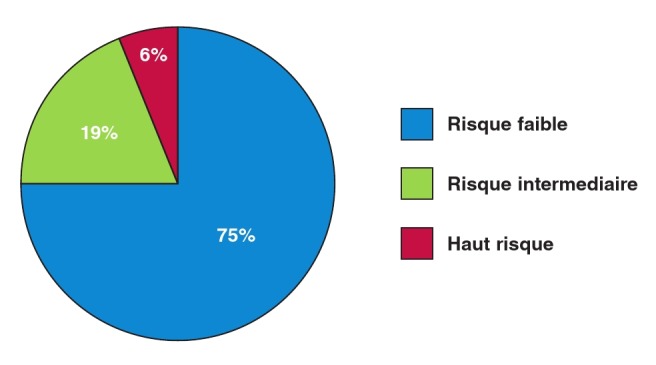
Répartition des patients en fonction du risque de survenue de mort subite dans les 5 ans

Douze patients (75%) avaient un risque de survenue de mort subite faible (score < 4%), trois (18.75%) avaient un risque intermédiaire (> 4% et < 6%) et un (6.25%) avait un haut risque (> 6%) selon le score en ligne de l’ESC (Fig. 3).

Treize patients étaient sous traitement à base de bétabloquant et d’inhibiteur calcique. Une patiente avait eu un DAI avec fonction pace maker. Il s’agit d’une patiente de 51 ans qui avait des antécédents de syncopes, d’accident ischémique transitoire et de mort subite dans la famille. Elle présentait un gradient maximal intra-VG de plus de 40 mm Hg au repos (haut risque de mort subite) et des troubles de la conduction au Holter ECG (alternance BAV II type I et type II). Un patient de 27 ans qui avait un gradient maximal intra-VG > 50 mmHg aux efforts minimes et une mauvaise adaptation de la TA à l’effort (risque intermédiaire) a été proposé à la myectomie.

## Discussion

La MS constitue la hantise au cours de la CMH. Les principaux facteurs de risque de MS reconnus sont:^1^,^8^,^9^

- âge jeune au moment du diagnostic (moins de 30 ans)
- histoire familiale de CMH avec mort subite prématurée
- syncopes répétées (à l’effort ou inexpliquée)
- réponse anormale de la pression artérielle à l’effort (différence pression artérielle au maximum de l’effort et pression artérielle (PA) de repos < 20–25 mmHg, surtout avant 40 ans)
- tachycardie ventriculaire (TV) non soutenue (surtout si répétée ou prolongée)
- arrêt cardiaque récupéré
- TV soutenue
- hypertrophie importante (paroi ≥ 30 mm)
- mutation maligne (celles du gène troponine T, R403Q du gène MYH7).

Dans notre étude, nous avions retrouvé une prédominance masculine avec 68% d’hommes. Niamkey à Abidjan^10^ retrouvait une prédominance masculine à 66.7% dans une étude ayant porté sur l’identification des facteurs majeurs de MS parmi les patients (au nombre de six) suivis pour CMH. L’âge moyen de nos patients était de 53.2 ans avec des extrêmes de 27 et 79 ans. Niamkey^10^ retrouvait un âge moyen de 30.5 ans avec des extrêmes de cinq et 45 ans. Maron^11^ retrouvait un âge moyen de 34 ans dans une étude en 1981. La différence d’âge de notre étude et celles des deux auteurs est due au choix des âges de leurs échantillons.

Trois patients présentaient des troubles du rythme ventriculaire au Holter ECG, dont un cas d’hyperexcitabilité ventriculaire degré V de Lown. Une patiente présentait des troubles de la conduction à type d’alternance de BAV de 2ème degré Mobitz I et Mobitz II sur fond de nombreux épisodes de bradycardie.

Le plus jeune de nos patients (27 ans) a présenté une mauvaise adaptation des chiffres tensionnels à l’effort. Cette désadaptation tensionnelle à l’effort, les troubles du rythme ventriculaire et de la conduction sont des facteurs qui font la gravité de la maladie. Ils sont à rechercher lors de l’évaluation initiale du patient à la recherche de facteurs pouvant favoriser la survenue de MS. Charron^12^ retrouvait une hyperexcitabilité supraventriculaire et notait aussi une TV non soutenue chez 25% des patients adultes, les TV soutenues étant rares. Il faisait ressortir la réponse anormale de la PA à l’effort, surtout avant 50 ans comme un facteur de risque de survenue de mort subite. Toutefois, un seul des facteurs de risque majeurs n’a que peu de valeur prédictive positive (environ 15–20%). C’est l’association de plusieurs d’entre eux qui doit être considérée.^12^,^13^

Concernant le risque de MS, Niamkey^10^ retrouvait 33.3% de haut risque de décès et un risque intermédiaire chez les autres patients. Maron et Mckenna^14^,^15^ notaient un taux de MS évalué entre 2 à 3% par an chez les adultes et entre 4 à 6% par an chez les enfants. Toutefois, ces travaux présentaient des biais de sélection importants. Cannan, Cecchi et Kofflard^16^-^18^ dans des études plus récentes réalisées dans des populations moins sélectionnées ont retrouvé des taux de MS bien inférieurs. Ils étaient de l’ordre de 1 à 2% par an, et même moins pour certaines études, indiquant que le pronostic des CMH est bien meilleur que celui communément admis.^19^

Il est à noter que ces travaux ont évalué l’évolution et le pronostic de la maladie tandis que notre étude a évalué le risque de survenue de MS dans les cinq ans en utilisant le calculateur en ligne de l’ESC. Ce dernier permet de détecter les patients à même d’être proposés à l’implantation d’un DAI. Ce calculateur présente toutefois certaines limites:^20^ il n’est pas utilisable chez le patient de moins de 16 ans, l’athlète de compétition d’élite, le sujet ayant subi une alcoolisation septale ou une myectomie, de même que lorsque l’hypertrophie septale maximale est ≥ 35 mm. En plus, il n’intègre pas les données concernant le test d’effort. C’est d’ailleurs ce qui explique qu’un de nos patients qui avait une désadaptation tensionnelle à l’effort (facteur de risque majeur) a eu un risque quotté intermédiaire.

La prise en charge intégrait, en plus des règles d’hygiène de vie et de proscription de sport de compétition, le traitement médical à base de bétabloquant et d’inhibiteur calcique bradycardisant (chez 13 patients). La patiente à haut risque de survenue de MS et qui présentait le trouble de la conduction avait bénéficié d’un DAI avec fonction pace maker. Elle est en instance de traitement complémentaire à type d’alcoolisation septale voire de myectomie vu la persistance de sa symptomatologie et du gradient intra-VG.

La prise en charge de la CMH fait appel à deux grandes catégories de traitement: celui médicamenteux et celui non médicamenteux.^21^ Le traitement médical est de première intention et fait appel classiquement aux bétabloquants, vérapamil et disopyramide (réservé aux cas résistants aux deux premiers chez les patients avec obstruction).^22^ Les autres molécules sont administrées en fonction du tableau clinique du patient avec une prudence accrue pour l’utilisation des diurétiques.

Le traitement non médicamenteux est réservé aux patients ayant un gradient intra-VG résistant au traitement médical et aux patients restant symptomatiques malgré un traitement optimal. Il s’agit de la myotomie-myectomie de Marrow, de l’alcoolisation septale, de la stimulation double chambre et du DAI avec ou sans fonction pacemaker. Selon les recommandations, la prévention nécessite une évaluation du risque de MS. Ainsi, un DAI devrait être implanté à tout patient à haut risque de MS.^23^

## Conclusion

L’évaluation du risque de survenue d’une cardiomyopathie hypertrophique est une étape cruciale permettant d’avoir un traitement adéquat. Notre travail portant sur l’évaluation du risque de mort subite dans la cardiomyopathie hypertrophique à Dakar a trouvé une prédominance de risque faible et intermédiaire de mort subite à cinq ans. Le haut risque n’existait que dans 1 cas. L’évaluation de ce score a par ailleurs, permis de proposer une prise en charge adéquate chez nos patients.
